# DISENTANGLE THE RELATION BETWEEN FRAILTY, SELF-RATED HEALTH, AND DEATH: IMPLICATIONS FOR RESILIENCE

**DOI:** 10.1093/geroni/igad104.1450

**Published:** 2023-12-21

## Abstract

Frail older adults represent a heterogeneous group. Identifying factors predictive of adverse outcomes among this group could advance our understanding of ways to withhold the devastating effects of frailty. Self-rated health (SRH) is a valuable health measure among the general population. However, it is unknown whether SRH remains predictive among the frail. We examined the joint association of frailty and SRH with mortality and assessed the association between SRH and death among the frail. Data were from the UK Biobank, a large-scale cohort study comprising 0.5 million individuals. At baseline, participants were assessed for physical frailty (non-frail, prefrail, and frail) and SRH (excellent/good, fair, and poor). The Poisson model was used to address the study’s aims. Age, sex, education, marital status, and smoking were adjusted. Of 488,590 participants, 3.2% were frail, and 4.3% reported poor health. Although frail participants were more likely to report poor health, 19.7% of the frail reported excellent/good health, and 15.9% of the non-frail reported fair or poor health. SRH was strongly associated with death among the non-frail, prefrail, and frail subgroups. Among frail participants, reporting fair and excellent/good was associated with a 17% and 25% lower hazard of death. The association between SRH and death was stronger among the frail than the non-frail, although the interaction term was insignificant. Future investigation into the determinants of positive SRH despite being frail could provide new targets for intervention to improve resilience and risk screening of downstream outcomes in the most vulnerable.

